# Supplementary material for: All-Atom Molecular Dynamics Simulations of Dimeric Lung Surfactant Protein B in Lipid Multilayers
Source: Int J Mol Sci. 2019 Aug 8;20(16):3863. doi: 10.3390/ijms20163863 (PMC6719169; doi:10.3390/ijms20163863)
Supplement: Supplementary file 1 [file ijms-20-03863-s001.pdf]

International Journal of
Molecular Sciences

Supplementary Figures

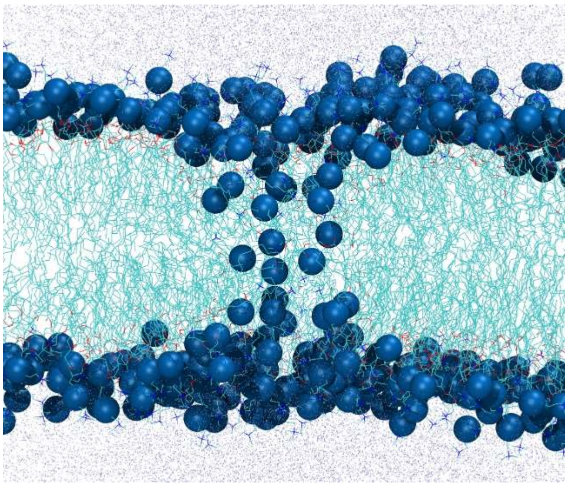

Figure S1. Snapshot of a preformed pore in a POPC lipid bilayer. Phosphorous atoms in lipid headgroups are shown as blue spheres.

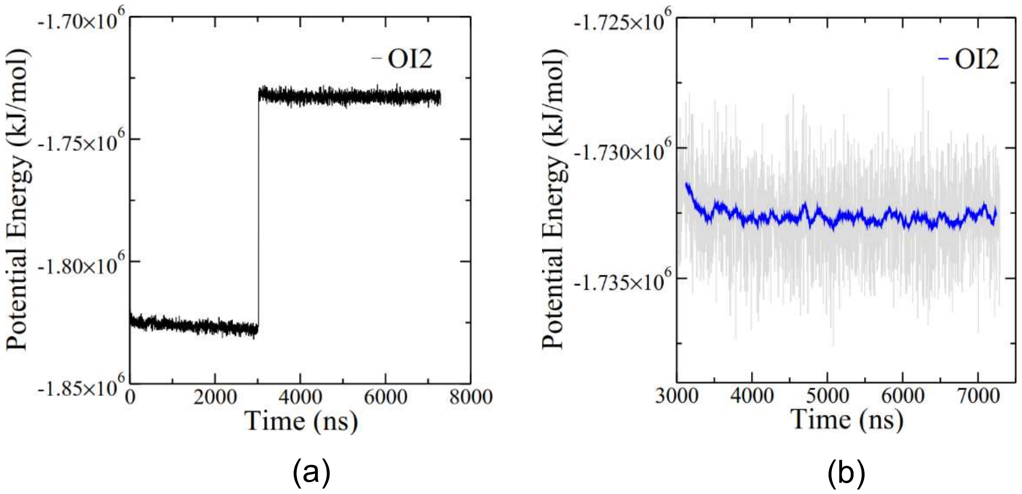

Figure S2. Potential energy for the OI2 simulation, shown for (**a**) the full simulation (temperature was increased from 310K to 340K at 3 μs), and (**b**) the high temperature segment of the simulation alone. In (**b**), the blue line represents a running average over 50 points while grey indicates individual time points (spaced 2 ns apart).

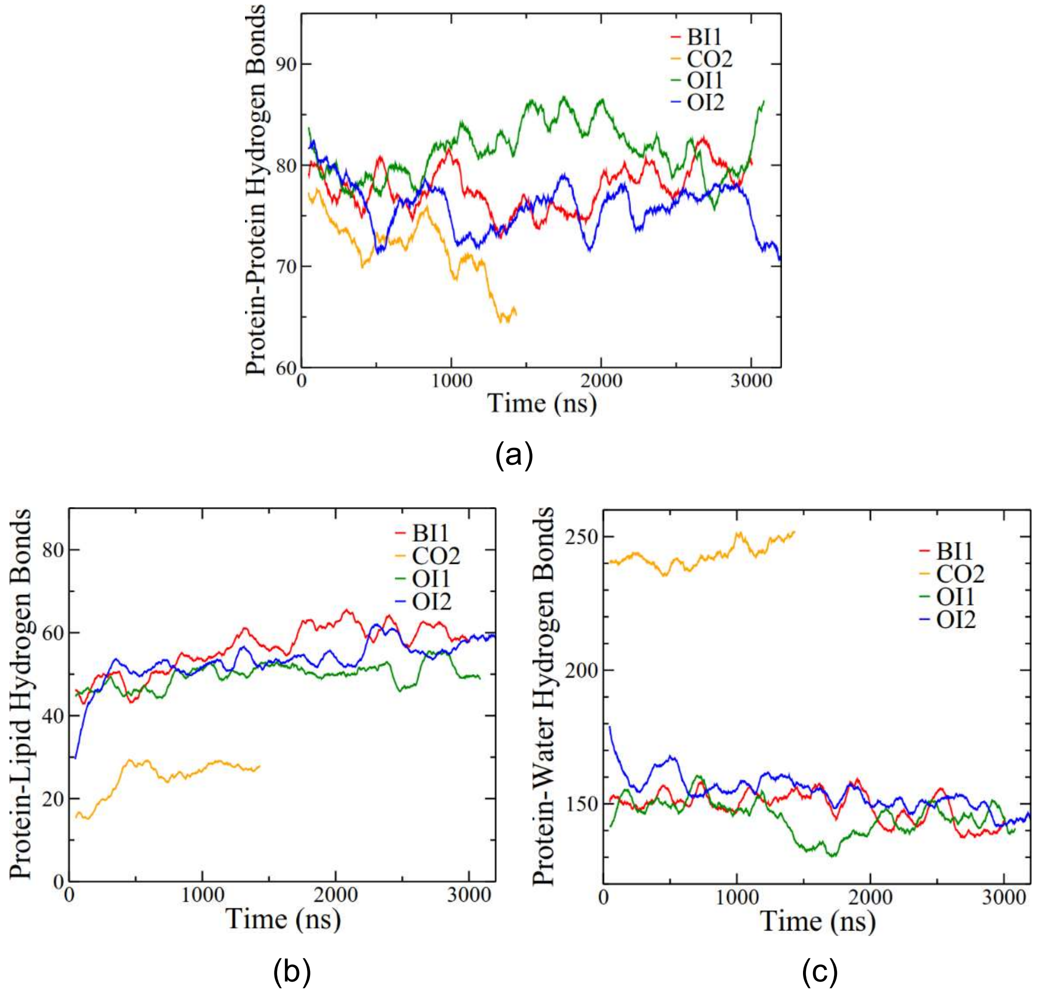

Figure S3. The progression of hydrogen bonding in the four simulations, showing (**a**) the number of internal hydrogen bonds within the protein, as well as the number of hydrogen bonds present between (**b**) protein and lipid, and (**c**) protein and water. The number shown in (**a**) includes helical hydrogen bonds. Hydrogen bonds between the two subunits were exceedingly rare in all systems. For the sake of comparison, only the 310K portion of the OI2 simulation is shown. Running averages over 50 points.

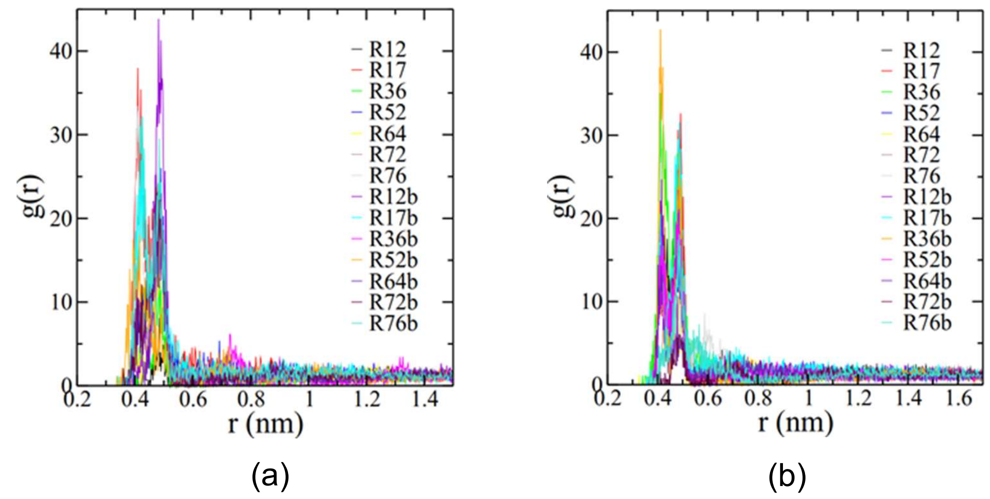

Figure S4. Radial distribution function of phosphorus atoms in lipids to the terminal side-chain carbon in arginine residues averaged over the last half of the simulation for (**a**) BI1 and (**b**) OI1.

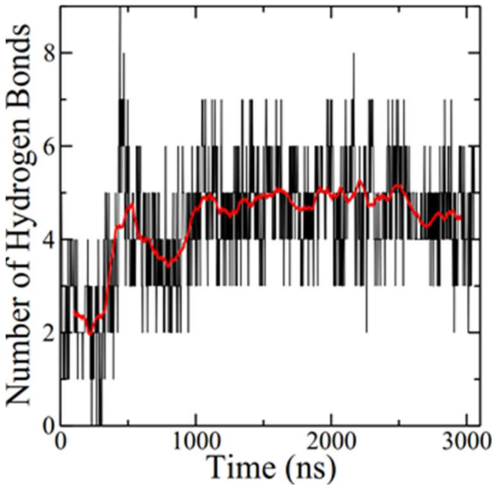

Figure S5. Hydrogen bonding between R36 residues from both subunits, located at the centre of the bilayer, and water over the course of the BI1 simulation.

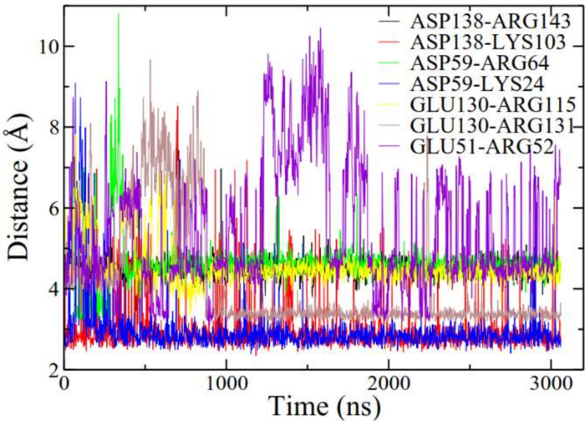

(a)

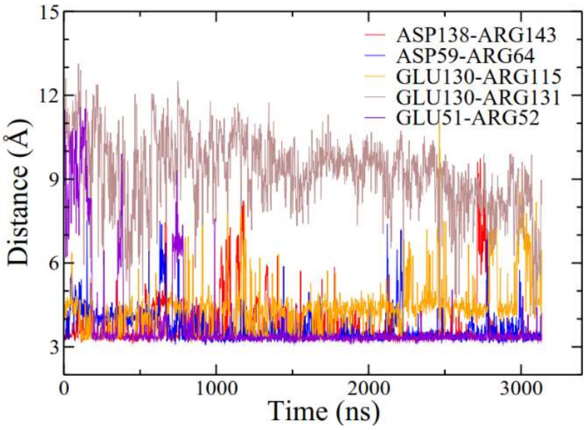

(b)

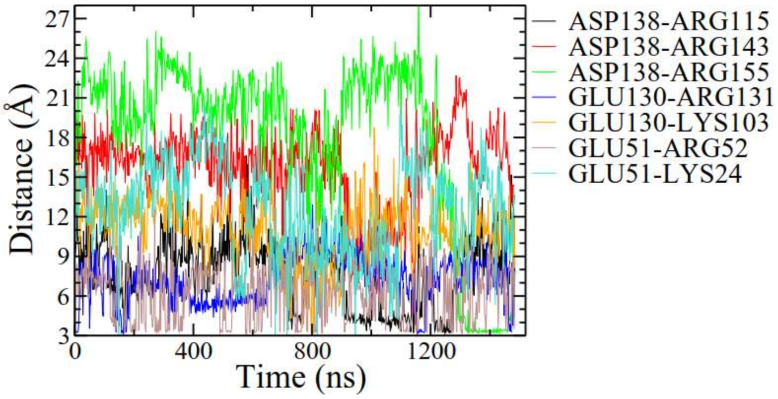

(c)

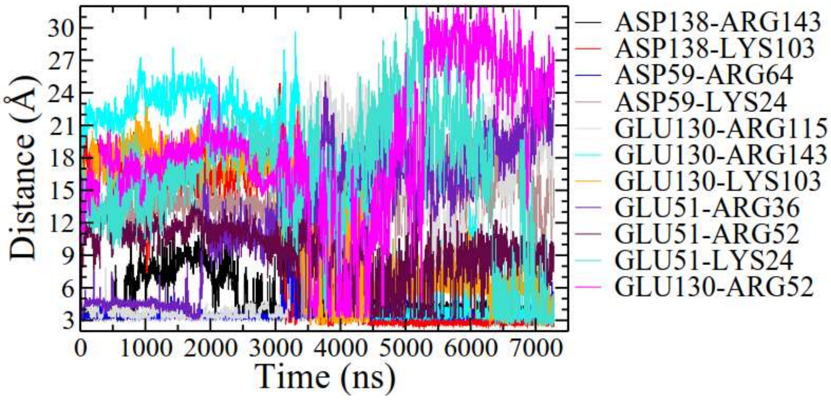

(d)

Figure S6. Salt bridging patterns in (**a**) BI1, (**b**) OI1, (**c**) CO2 and (**d**) OI2 over the course of each simulation. The y-axis plots the distance between charged sidechains. Only pairs of residues whose charged atoms were found within 3.2 Å of each other at some point during the simulation are plotted. In the legends, residues of one subunit are numbered as 1 to 79 and the other subunit is numbered as 80 to 158. Inter-subunit salt bridging was only seen in the OI2 simulation, occurring transiently after temperature was increased.
